# Polarization of Low-Grade Inflammatory Monocytes Through TRAM-Mediated Up-Regulation of Keap1 by Super-Low Dose Endotoxin

**DOI:** 10.3389/fimmu.2020.01478

**Published:** 2020-07-16

**Authors:** Allison Rahtes, Liwu Li

**Affiliations:** Department of Biological Sciences, Virginia Tech, Blacksburg, VA, United States

**Keywords:** monocyte polarization, subclinical dose endotoxin, low grade inflammation, Keap1, TRAM

## Abstract

Subclinical endotoxemia [low levels of bacterial endotoxin (LPS) in the blood stream] has been correlated with chronic inflammatory diseases, with less-understood mechanisms. We have previously shown that chronic exposure to super low doses of LPS polarizes monocytes/macrophages to a pro-inflammatory state characterized by up-regulation of pro-inflammatory regulators such as p62 and simultaneous down-regulation of anti-inflammatory/resolving regulators such as Nrf2. Building upon this observation, here we show that chronic exposure to super-low doses of LPS leads to accumulation of the Nrf2-inhibitory protein Keap1 in murine monocytes. This is accompanied by increases of p62 and MLKL, consistent with a disruption of autolysosome function in polarized monocytes challenged by super-low dose LPS. Monocytes subjected to persistent super-low dose LPS challenge also accumulate higher levels of IKKβ. As a consequence, SLD-LPS challenge leads to an inflammatory monocyte state represented by higher expression of the inflammatory marker Ly6C as well as lower expression of the anti-inflammatory marker CD200R. Further analysis revealed that Keap1 levels are significantly enriched in the Ly6C^hi^ pro-inflammatory monocyte population. Finally, we show that the TLR4 signaling adaptor TRAM is essential for these effects. Together our study provides novel insight into signaling mechanisms behind low-grade inflammatory monocyte polarization unique to chronic super-low dose LPS exposure.

## Introduction

Emerging studies reveal dynamic programming processes of innate leukocytes by varying dosages of bacterial endotoxin Lipopolysaccharide (LPS) (1–3). Prolonged challenges of monocytes/macrophages with higher doses LPS (100 ng−1 μg/mL) lead to a hyporesponsive state termed “endotoxin tolerance.” In contrast, monocytes/macrophages persistently challenged with subclinical low-dose LPS (100 pg−1 ng/mL) are programmed into a low-grade inflammatory state characterized by sustained expression of pro-inflammatory mediators (4). Subclinical low-dose LPS (SLD-LPS) was shown to be present in humans with chronic inflammatory complications such as chronic infection, obesity, and aging (5–7). Despite its clinical significance, the molecular mechanisms underlying the low-grade inflammatory polarization of monocytes under persistent SLD-LPS challenge are not well-understood.

At the mechanistic level, monocytes/macrophages challenged with prolonged high doses of LPS have shown increased compensatory resolving processes including autolysome-mediated degradation of p62 and IKKβ (8) as well as the upregulation of anti-oxidative responses through the induction of Nrf2 (9). In contrast, we recently reported that monocytes persistently challenged with SLD-LPS accumulate the pro-inflammatory signaling mediator p62, due to the disruption of autophagosome fusion with lysosome (10). SLD-LPS treatment also reduces the activity of the anti-inflammatory mediator Nrf2, which may collectively contribute to the establishment of non-resolving low-grade inflammatory state (11). However, mechanisms for Nrf2 inactivation by SLD-LPS are still unclear. Nrf2 is under the control of Keap1 which serves as the cul3-ubiquitin E3 ligase adaptor that targets Nrf2 for inactivation (12). Cells challenged with high dose LPS exhibit reduced Keap1 levels, potentially through lysosome-mediated degradation, which correlates with elevated Nrf2 and anti-inflammatory compensation (13). However, the regulation of Keap1 in monocytes/macrophages challenged with SLD-LPS has not been characterized. Given the knowledge gap regarding the effects of SLD-LPS, our current study will focus on examining the effects of SLD-LPS on Keap1 regulation in primary monocytes.

LPS signals through Toll-Like-Receptor 4 (TLR4) and utilizes multiple adaptor molecules such as TIRAP-MyD88 and TRAM-TRIF to initiate and sustain cellular activation (14). Traditionally, the MyD88-dependent signaling pathway was associated with activation of NF-κB and pro-inflammatory mediators while the TRAM/TRIF pathway is associated with interferon production (15). However, emerging studies suggest that the TRAM/TRIF pathway also plays a key role in TLR4-induced NF-kB inflammatory activation (16–19), potentially through sustaining a prolonged inflammatory response as opposed to the transient MyD88-dependent response (18). Consistent with these observations, we reported that TRAM is preferentially involved in the low-grade inflammatory polarization of cultured murine macrophage cells by SLD-LPS (20). Here, we aimed to further test the role of TRAM during the regulation of Keap1 and related inflammatory signaling processes in monocytes challenged with SLD-LPS.

Using primary bone marrow monocytes harvested from WT and TRAM deficient mice, we examined the regulation of Keap1 through independent analysis of immuno-blot and flow cytometry. We further tested the levels of molecules related to disrupted autolysosome function such as p62 and MLKL in WT and TRAM deficient monocytes challenged with SLD-LPS. Accumulation of p62 and Keap1 due to the disruption of autolysosome function can further lead to the induction of IKKβ (8, 21–23). We therefore further examined the levels of IKKβ in WT and TRAM deficient monocytes challenged with SLD-LPS. We observed that SLD-LPS potently induces the accumulation of Keap1, p62, MLKL, IKKβ in WT monocytes, but not in TRAM deficient monocytes. Functionally, monocyte polarization comparing WT and TRAM deficient monocytes challenged with SLD-LPS was measured by flow cytometry for Ly6C as a key marker of inflammatory polarization as well as CD200R as a marker for anti-inflammatory polarization. We demonstrated that SLD-LPS preferentially drives the inflammatory polarization, as reflected by increased Ly6C and reduced CD200R, in WT but not TRAM deficient monocytes. Together, our current study reveals novel mechanisms regarding Keap1-mediated inflammatory polarization of low-grade inflammatory monocytes by subclinical dose LPS, critically dependent upon TRAM.

## Materials and Methods

### Mice and Cell Culture

All mice were housed in standard pathogen-free conditions and ethically euthanized according to protocols approved by the Virginia Tech Institutional Animal Care and Use Committee (IACUC) prior to start of the study. A combination of both male and female mice of C57BL/6 background ranging in age from 8 to 12 weeks were used for all studies. TRAM^−/−^ mice on C57BL/6 background were provided by Dr. Holger Eltzschig research group. Bone marrow cells were harvested from femur and tibia and cultured for 5 days with 10 ng/mL M-CSF (Peprotech #315-02) after red-blood cell lysis with ACK lysis buffer (ThermoFisher #A1049201). As described in our previous publication (24), cells were cultured for 5 days in the presence of either PBS, 100 pg/mL LPS (SLD-LPS), or 1 μg/mL LPS (High-dose LPS) in RPMI medium (Sigma #R8758) supplemented with 10% FBS (Fisher #SH3007003HI), 100 U/mL Penicillin/Streptomycin (ThermoFisher #15140122), and 2 mM L-Glutamine (Life Technologies #25030-081). Half of the media was removed and replaced with fresh media and treatments on days 2 and 4. Cultured cells harvested from naïve donor male or female mice responded similarly upon *in vitro* culture and challenge with LPS. LPS is derived from *E. coli* O111:B4 and supplied by Sigma (#L2630) and stored at −20°C as a stock solution at 1 mg/mL in PBS.

### Western Blot

Cell lysates were harvested from culture BMDMs with 2% SDS lysis buffer containing protease inhibitor cocktail (Sigma #P8340) and phosphatase inhibitor cocktails 2 (Sigma #P5726) and 3 (Sigma #P0044). Lysates were incubated on ice for 15 min, boiled at 95–100°C for 5 min, and then stored at −20°C before use in assay. Lysates were separated by SDS-PAGE followed by transferring to methanol-soaked PVDF membranes under ice for 2 h. Membranes were blocked in 5% non-fat milk for 1 h followed by incubation with the following primary antibodies respectively: Keap1 (Cell Signaling #8047), p62 (Cell Signaling #5114), MLKL (Cell Signaling #37705), IKK-β (Novus Biologicals # NB100-56509), phospho-NF-κB p65 (Ser536) (Cell Signaling # 3031S), total NF-κB p65 (D14E12) XP (Cell Signaling #8242S), GAPDH (Cell Signaling #2118), or beta actin (Cell Signaling #4970). Blots were incubated overnight or for 3 days before detection using HRP-linked Anti-rabbit IgG antibody (Cell Signaling #7074) and Advansta ECL detection kit (VWR #490005-020). Relative protein levels were quantified using ImageJ software. Graphpad Prism 6.0 software was used for statistical analysis and figure preparation as detailed in the statistical analysis section.

### qRT-PCR

RNA was isolated from cultured BMDMs using TRIzol (Fisher #15596018) following the manufacturer's protocol, and was reverse transcribed using the High-capacity cDNA reverse transcription kit (ThermoFisher #4368814) and the Eppendorf Mastercycler personal thermocycler. cDNA was diluted with nuclease-free water before use in the qPCR reaction. Sso Advanaced SYBR Green Supermix (BioRad #1725274) was used for the qPCR reaction performed on the BioRad CFX96 Touch Real-Time System. Results were then analyzed using the double delta CT method, with HPRT-1 as the house-keeping control gene, followed by normalization to the control sample (PBS-treated) gene expression where indicated. Primers were designed using NCBI RefSeq database and synthesized by IDT with standard desalting and 25 nM DNA Oligo preparation. Primers were diluted to a concentration of 10 μM in nuclease-free water prior to use in qPCR reaction. Primer sequences are as follows:

HPRT-1: F−5′-CTCAGACCGCTTTTTGCCG−3′; R−5′-CGCTAATCACGACGCTGGG−3′

Keap1: F−5′-CTCAACCGCTTGCTGTATGC−3′; R−5′-CATAGCCTCCGAGGACGTAG−3′

NQO1: F−5′- CCATGTACGACAACGGTCCT−3′; R−5′-CGCAGGATGCCACTATGAA−3′

HMOX1:F−5′-TTGAGGAGCTGCAGGTGATG−3′;R−5′-GCAGTATCTTGCACCAGGCT−3′

Catalase:F−5′-CAGTCAGGTGCGGACATTCT−3′;R−5′-CAGGGTGGACGTCAGTGAAA-3′

### Protein/Transcript Analysis

Protein quantification values normalized to the house-keeping control from Western blot data were divided by relative transcript values as determined by the ΔΔCT method. Graphpad Prism 6.0 software was used to perform statistical analyses and prepare the figure illustration.

### Co-immunoprecipitation

Co-immunoprecipitation experiments were performed with lysates from cultured BMDMs following the immunoprecipitation protocol provided by the manufacturer Abcam Inc. In brief, cell lysates were harvested on ice in non-denaturing lysis buffer (20 mM Tris HCl pH8, 137 mM NaCl, 1% Triton X-100, and 2 mM EDTA in DI water) containing PIC (Sigma #P8340). Lysates were incubated under gentle agitation for 30 min. at 4°C following by centrifugation. Protein concentrations were determined using the BioRad DC Protein Assay kit (#5000112) and a BSA standard. 50 μg of lysate from each treatment was added to a tube containing Protein G Agarose beads (Active Motif #37449) linked to antibody, and incubated under gentle agitation overnight. 50 μL of Protein G Agarose bead slurry was incubated with 5 μg of antibody under gentle agitation at 4°C for 4 h and washed in lysis buffer prior to addition of lysate. Antibodies against Nrf2 (ThermoFisher #PA5-27882) and Keap1 (ProteinTech #10503-2-AP) were used. Beads were washed the following day and complex eluted in SDS Buffer and boiled before Western blot. Blots were probed with the opposite antibody (Nrf2 on Keap1 IP blot and Keap1 on Nrf2 IP blot). Blots were detected using a conformation-specific anti-rabbit HRP-linked IgG secondary antibody (Cell Signaling #5127) and Advansta ECL detection kit.

### Flow Cytometry

Cultured BMDMs were harvested on ice, centrifuged at 300 g for 5 min at 4°C and re-suspended in Hanks Balanced Salt Solution (HBSS) containing 2% FBS for cell counting. Approximately 2 X 10^5^ cells were added to each well of a 96-well flat-bottomed plate and blocked on ice for 15 min in Fc block antibody solution before staining for surface antibodies (FITC-Ly6C and APC-CD200R) for 40 min at 4°C in the dark. After 3 × washing with PBS, cells were stained with fixable viability dye eFluor™ 780 (ThermoFisher #65-0865) at 1:1,000 in PBS for 30 min at 4°C in the dark, followed by washing once with FACS buffer. Samples were then fixed, permeabilized, and stained for intracellular Keap1 using the BD Transcription Factor Phospho Buffer set (Fisher #NC1023103) and PE-Keap1 antibody following the manufacturer's protocol. Flow cytometry was performed and compensation settings adjusted using BD FACS Canto II and BD FACS DIVA software. FlowJo software version 10 was used for gating and analysis, while Graphpad Prism software 6.0 was used for statistical analysis of MFI and to generate representative bar graphs of MFI. The following antibodies were used for flow cytometry: FITC-Ly6C (BioLegend #128005), APC-CD200R (eBioscience #17-5201-82), and PE-Keap1 (Novus Biologicals #NBP2-71496PE). All antibodies were used at 1:200 dilution in FACS buffer except for the PE-Keap1 which was at 1:200 in Perm/Wash Buffer.

### Statistical Analysis

All data are representative of at least 3 independent experiments harvested from three different mice. Graphpad Prism software 6.0 was used to perform statistical analysis in the form of either an unpaired student *t*-test (for PBS vs. SLD-LPS comparisons) or one-way ANOVA followed by Tukey's multiple comparisons for statistical analysis between groups. ^*^p < 0.05, ^**^p < 0.01, ^***^p < 0.001, and ^****^p < 0.0001. Detailed statistical methods and experimental repeats were noted in the respective figure legends.

## Results

### Keap1 Accumulates in Monocytes Persistently Challenged With Super-Low Dose LPS

Using an immortalized mouse macrophage cell line, we previously published the non-resolving pro-inflammatory polarization of mouse macrophages by SLD-LPS exposure characterized in part by increased levels of the ubiquitinated (inactive) form of the homeostatic transcription factor Nrf2 and decreased transcript levels of one Nrf2's target genes, *slc40a1* (11). To further explore the molecular mechanism behind this phenomenon we examined the effects of chronic SLD-LPS exposure on Nrf2's regulatory protein Keap1 using our previously developed chronic exposure 5-days bone-marrow-derived monocytes (BMDMs) model, which mimics the establishment of a non-resolving low-grade inflammatory state (20).

**Figure 1:**
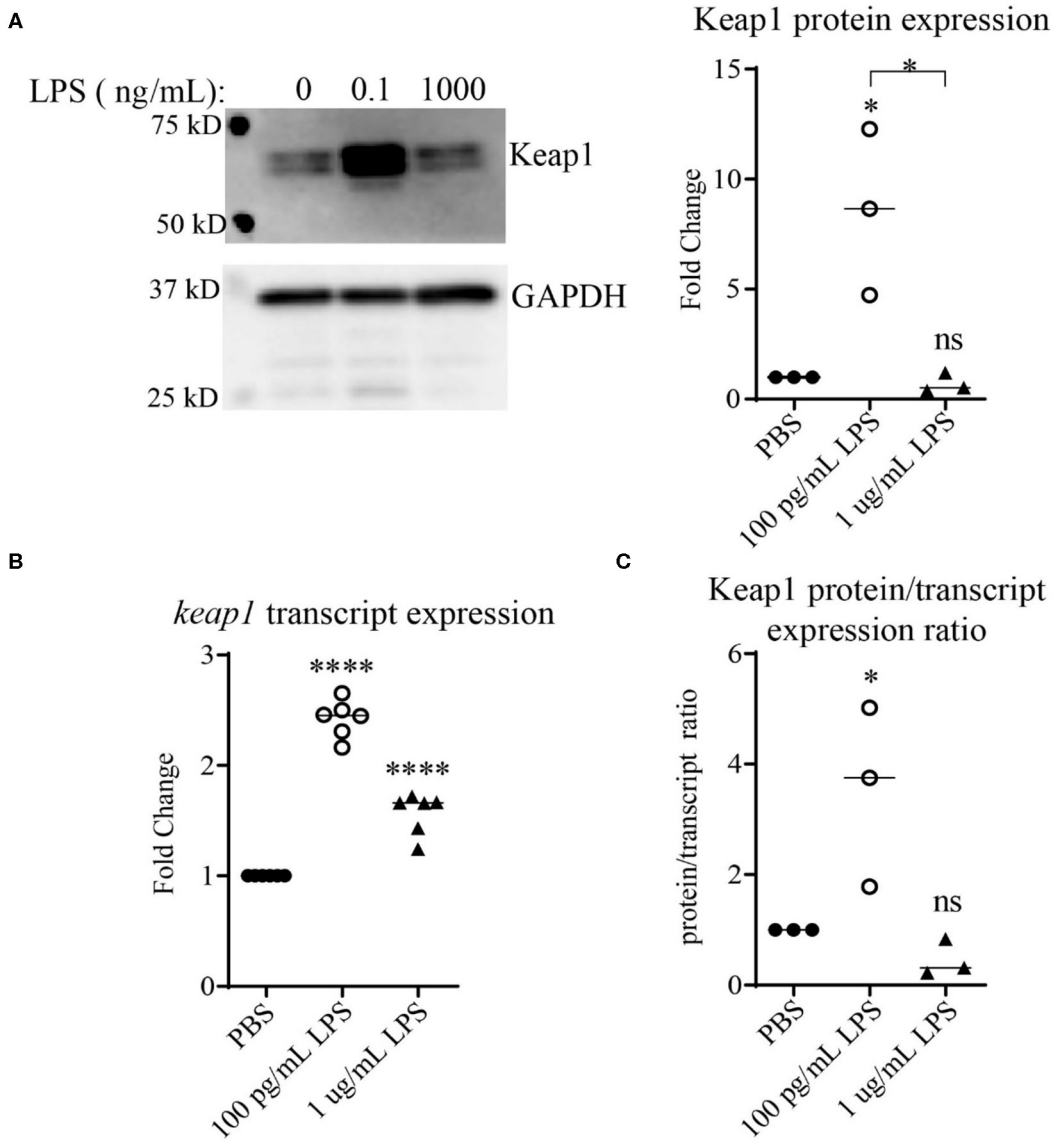
Chronic SLD-LPS leads to Keap1 protein accumulation. **(A)** Representative Western blot image (left panel) and quantification (right panel) of Keap1 expressions in BMDMs treated with the indicated doses of LPS or PBS for 5 days (*n* = 3). **(B)** *keap1* relative transcript levels as assessed by qRT-PCR using the ΔΔCT method (*n* = 5). **(C)** The quantified protein expression values in **(A)** divided by the transcript values in **(B)**. Data is representative of at least 3 separate experiments with cells cultured *in vitro* from three independent mice. ANOVA analysis with Tukey's multiple comparisons test. *p < 0.05, ****p < 0.0001.

Keap1 is a protein that under non-stress conditions binds Nrf2 in the cytosol, preventing its nuclear translocation and targeting it for ubiquitin-mediated degradation (25, 26). It has been shown that stimulation with high doses of LPS leads to activation of Nrf2 with decreased levels of Keap1 (13). To examine the effects of LPS concentration on Keap1, we first performed an initial pilot study using various dosages of LPS (Supplemental Figure S1). Higher doses of LPS led to some decrease in Keap1 (Supplemental Figure S1), consistent with the previous finding (13). In contrast, we observed that cells treated with chronic SLD-LPS (100 pg/mL) had the opposite effect, exhibiting a significant increase in Keap1 expression as compared to cells treated with PBS or high-dose (1 μg/mL) LPS (Figure 1A) To further determine whether this novel Keap1 accumulation was due to effects at the protein or transcript level, we performed qRT-PCR to assess the effects of chronic SLD-LPS on *keap1* transcription and observed that SLD-LPS lead to a significant increase in *keap1* mRNA levels, which was also observed (to a lesser degree) in high-dose LPS treated cells (Figure 1B). Integrating these two readouts, we divided the protein expression values (normalized to beta actin expression) by the transcript expression values to yield a protein/transcript expression ratio. As seen in Figure 1C, chronic SLD-LPS treated cells had a greater protein/transcript ratio than control-treated or high-dose LPS-treated cells, suggesting that while some of the increase is due to upregulation at the transcript level, the observed increase in Keap1 expression by SLD-LPS is primarily due to an increase at the protein level (Figure 1C).

### Chronic SLD-LPS Results in the Accumulation of p62 and MLKL

**Figure 2:**
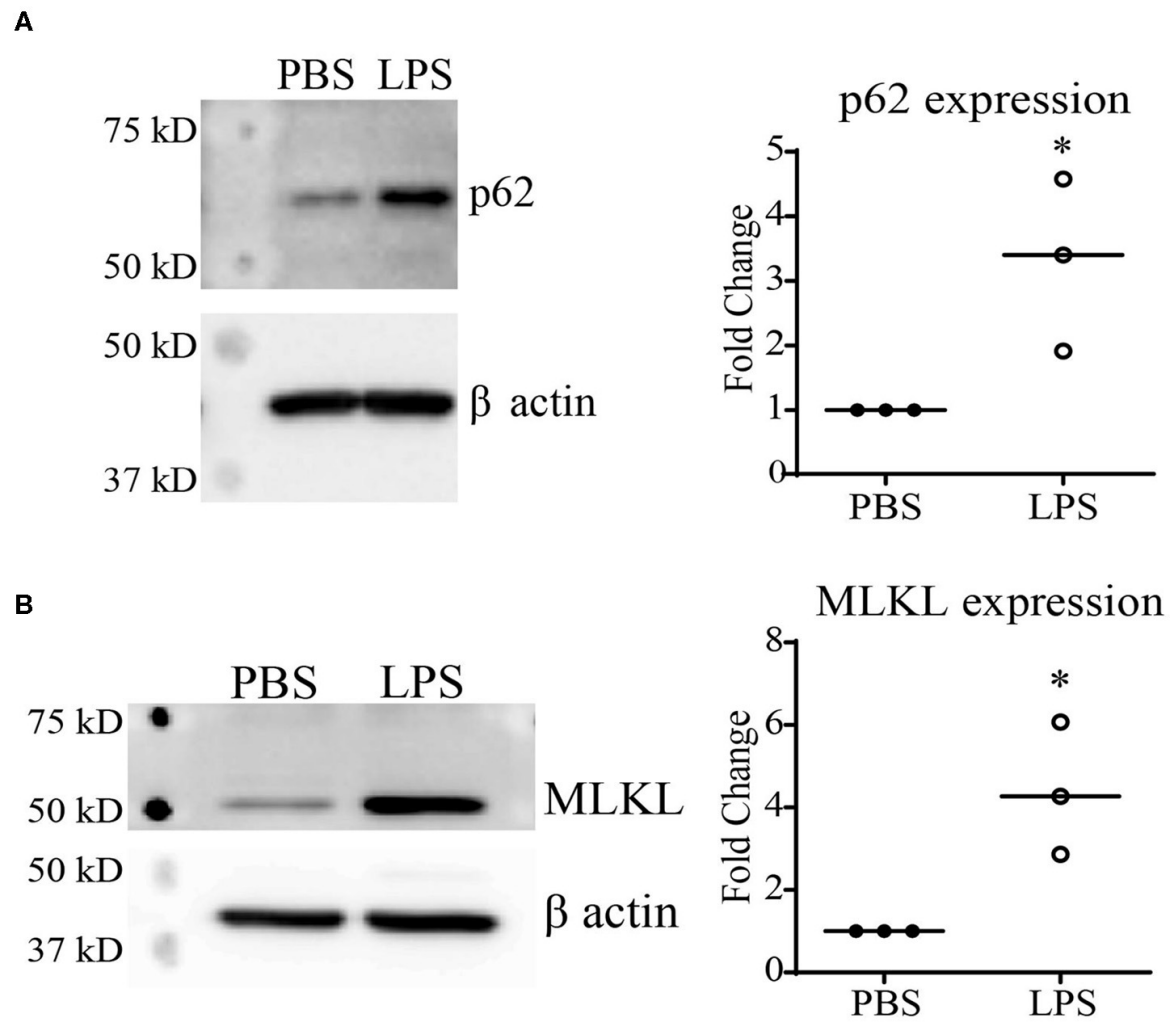
Chronic SLD-LPS treatment leads to the accumulation of p62 and MLKL. **(A)** Representative Western blot image (left panel) and quantification (right panel) of p62 expressions in BMDMs treated with the SLD-LPS (100 pg/mL) or PBS for 5 days (*n* = 3). **(B)** Representative Western blot image (left panel) and quantification (right panel) of MLKL expressions in BMDMs treated with the SLD-LPS or PBS for 5 days (*n* = 3). Data are representative of at least 3 separate experiments with cells cultured *in vitro* from three independent mice. Unpaired student *t*-test statistical analysis. *p < 0.05.

Previous reports using autophagy and proteasome inhibitors have suggested that Keap1 is primarily degraded through autophagy (13, 27), which is further confirmed through studies using Atg7^−/−^ mice (28). Our previous studies demonstrated that chronic SLD-LPS exposure can lead to a defect in lysosomal fusion, the final stage of autophagy (10, 29). Thus, we further tested the hypothesis that impaired autolysosomal degradation may be associated with SLD-LPS mediated Keap1 accumulation by testing the levels of two proteins (p62 and MLKL) associated with the disruption of lysosomal fusion. p62 (also known as sequestosome1) is a protein known to be involved in diverse processes such as NF-κB signaling (23, 30, 31), autophagy (32–34), and Nrf2 regulation (27, 35, 36). Importantly, p62 accumulation is a well-known marker for defective lysosomal fusion (34, 37, 38). We observed that chronic SLD-LPS challenge led to a significant increase in the levels of p62 protein (Figure 2A). We further examined the effects of SLD-LPS on MLKL, a protein capable of blocking autophagic completion through disrupting lysosomal membrane integrity (39, 40). As shown in Figure 2B, chronic SLD-LPS exposure resulted in significantly elevated levels of MLKL (Figure 2B). This supports our previous finding of disrupted lysosomal fusion by SLD-LPS and suggests that this may play a role in the SLD-LPS-mediated upregulation of Keap1.

### Increased Keap1 Expression Is Associated With Non-resolving Inflammatory Monocyte Polarization

**Figure 3:**
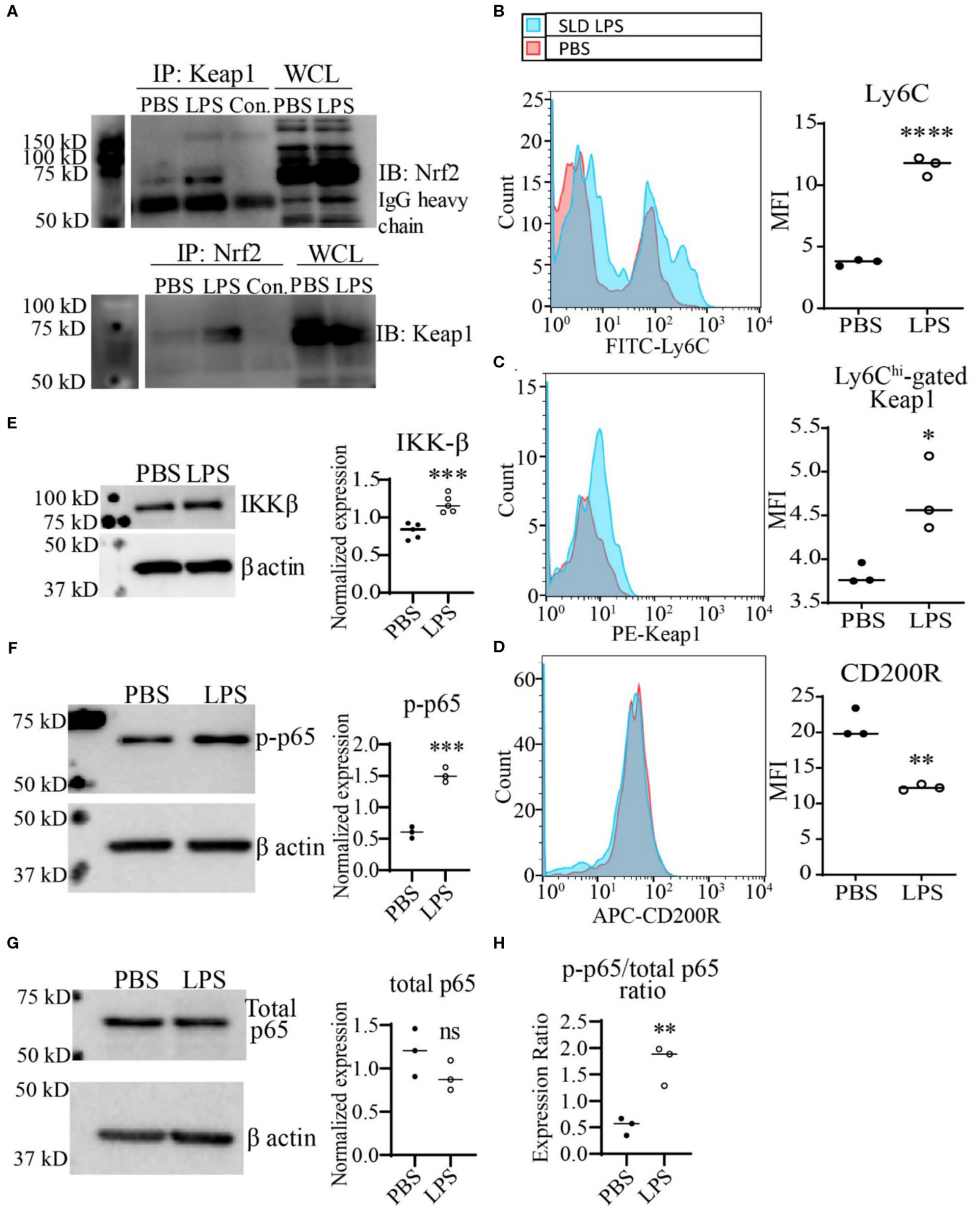
SLD-LPS-mediated Keap1 accumulation is correlated with a pro-inflammatory monocyte phenotype. BMDMs were treated with either PBS or SLD-LPS (100 pg/mL) for 5 days. **(A)** Representative Co-IP result with no lysate IP (just antibody and beads) (Con.) and the whole cell lysate input (WCL) controls. Keap1 antibody was used to pull-down Nrf2 (upper blot), and Nrf2 was used to pull down Keap1 (lower blot). See Supplemental Figures S3, S4 for un-altered blots. **(B–D)** The levels of Ly6C **(B)**, Keap-1 **(C)**, and CD200R **(D)** were analyzed and quantified using geometric mean fluorescence intensity (MFI) by flow cytometry. **(E–G)** Representative western blots of IKKβ **(E)**, p-p65 (S536) **(F)**, and total NF-κB p65 **(G)** in monocytes with quantification on the left. **(H)** p-p65/total p65 ratio. All data are representative of at least 3 separate experiments (*n* = 3, except for IKK-β where *n* = 5). Unpaired student *t*-test. *p < 0.05, **p < 0.01, ***p < 0.001, ****p < 0.0001.

Next, we further tested the functional relevance of Keap1 accumulation by SLD-LPS in monocytes As stated previously, Keap1 acts as an inhibitor of Nrf2 activity through direct interaction, leading to its cytosolic retention and inactivation. Therefore, we performed co-immunoprecipitation analysis in monocytes treated with SLD-LPS to test whether the accumulated Keap1 leads to inhibition of Nrf2 (Figure 3A). We observed that monocytes treated with SLD-LPS showed increased interaction between Keap1 and Nrf2, as compared to PBS-treated cells (Figure 3A). In addition, monocytes challenged with SLD-LPS failed to induce expression of the Nrf2-regulated genes *hmox1, nqo1*, and *catalase*, further confirming the SLD-LPS-mediated repression of Nrf2 activity (Supplemental Figure S2). Next, we tested the effect of Keap1 accumulation by SLD-LPS on classical markers of monocyte polarization such as the pro-inflammatory, M1-associated surface protein Ly6C (41, 42), and the anti-inflammatory, M2-associated marker CD200R (43), by flow cytometry (Figures 3B–D). We observed that chronic SLD-LPS challenge resulted in a significant increase in Ly6C expression (Figure 3B) which was correlated with increased Keap1 levels (Figure 3C). This was accompanied by a significant decrease in the expression of the anti-inflammatory marker CD200R (Figure 3D), suggesting that Keap1 accumulation is associated with non-resolving monocyte polarization by SLD-LPS.

To further examine the potential mechanism Keap1-mediated inflammatory monocyte polarization, we examined the levels of the NF-κB activating protein IKKβ. It was previously reported that Keap1 may associate with IKKβ, targeting it for autophagic degradation, and thereby leading to reduced NF-κB activity (8). Given our observations of increased expression of both Keap1 and p62, we tested whether persistent SLD-LPS treatment may similarly enhance accumulation of IKKβ. As shown in Figure 3E, monocytes persistently challenged with SLD-LPS exhibited a significant increase in the levels of IKKβ protein. Since IKKβ was shown to induce the phosphorylation of the p65 subunit of NF-κB at serine 536 (44), we further examined the status of p65-S536 phosphorylation in SLD-LPS treated monocytes. We observed that persistent SLD-LPS challenge led to a moderate, but significant increase in the levels of Ser536-phosphorylated p65 (Figure 3F). We further measured the total levels of p65 and observed no significant difference among PBS and SLD-LPS treated monocytes (Figure 3G). However, the ratio of p-p65 to total p65 was significantly increased in monocytes treated with SLD-LPS (Figure 3H). Together, our data suggest that chronic SLD-LPS challenge can lead to pro-inflammatory monocyte polarization through the accumulation of Keap1 and IKKβ.

### TRAM Is Necessary for SLD-LPS-Mediated Keap1 Accumulation

**Figure 4:**
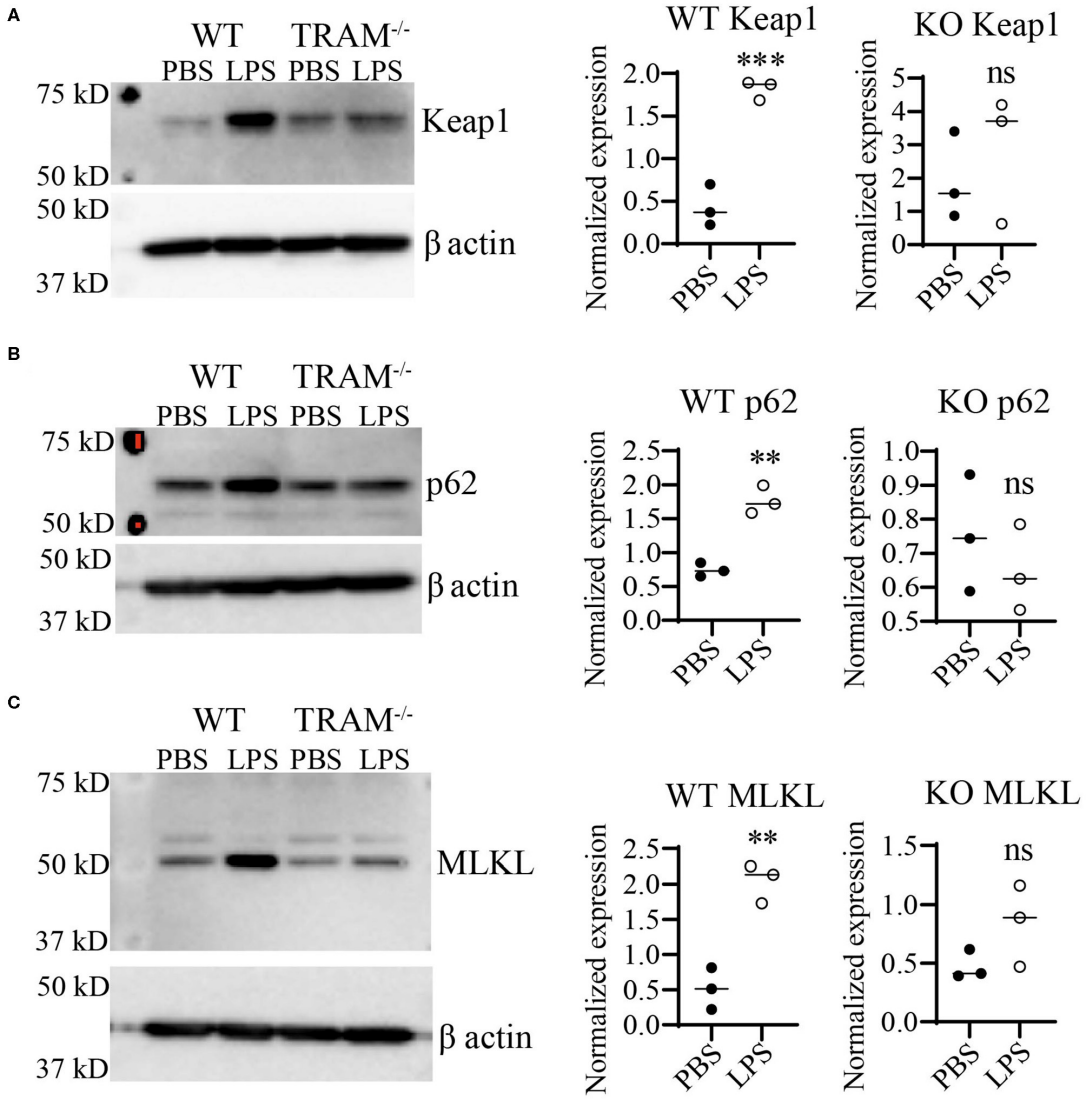
TLR4 adaptor TRAM is necessary for SLD-LPS-induced induction of Keap1, p62, and MLKL. Wild-type (WT) and TRAM^−/−^ (KO) BMDMs were treated with either PBS or SLD-LPS (100 pg/mL) for 5 days. **(A)** Representative Western blot image (left panel) and quantification (right panel) of Keap-1 expression in BMDMs (*n* = 3). **(B)** Representative Western blot image (left panel) and quantification (right panel) of p62 expression in BMDMs (*n* = 3). **(C)** Representative Western blot image (left panel) and quantification (right panel) of MLKL expression in BMDMs (*n* = 3). All data are representative of at least three independent experiments. unpaired student *t*-test. **p < 0.01, ***p < 0.001, ns, non-significant as compared to PBS group.

Preliminary data from our lab using immortalized cell lines suggested that the TRAM/TRIF TLR4 signaling pathway may be the preferred signaling pathway in response to chronic SLD-LPS (20). Further, the TRAM/TRIF pathway has been implicated in prolonged inflammatory responses (16, 18, 45), as well as the pathogenesis of the chronic inflammatory disease atherosclerosis (17). Thus, we tested whether TRAM may be responsible for the accumulation of Keap1 in monocytes persistently challenged with SLD-LPS. As shown in Figure 4, SLD-LPS failed to significantly increase the levels of either Keap1, p62, or MLKL in TRAM deficient monocytes (Figures 4A–C) as compared to WT monocytes, suggesting a key role for TRAM in the SLD-LPS-mediated accumulation of Keap1.

### TRAM Is Required for the Inflammatory Monocyte Polarization Induced by SLD-LPS

**Figure 5:**
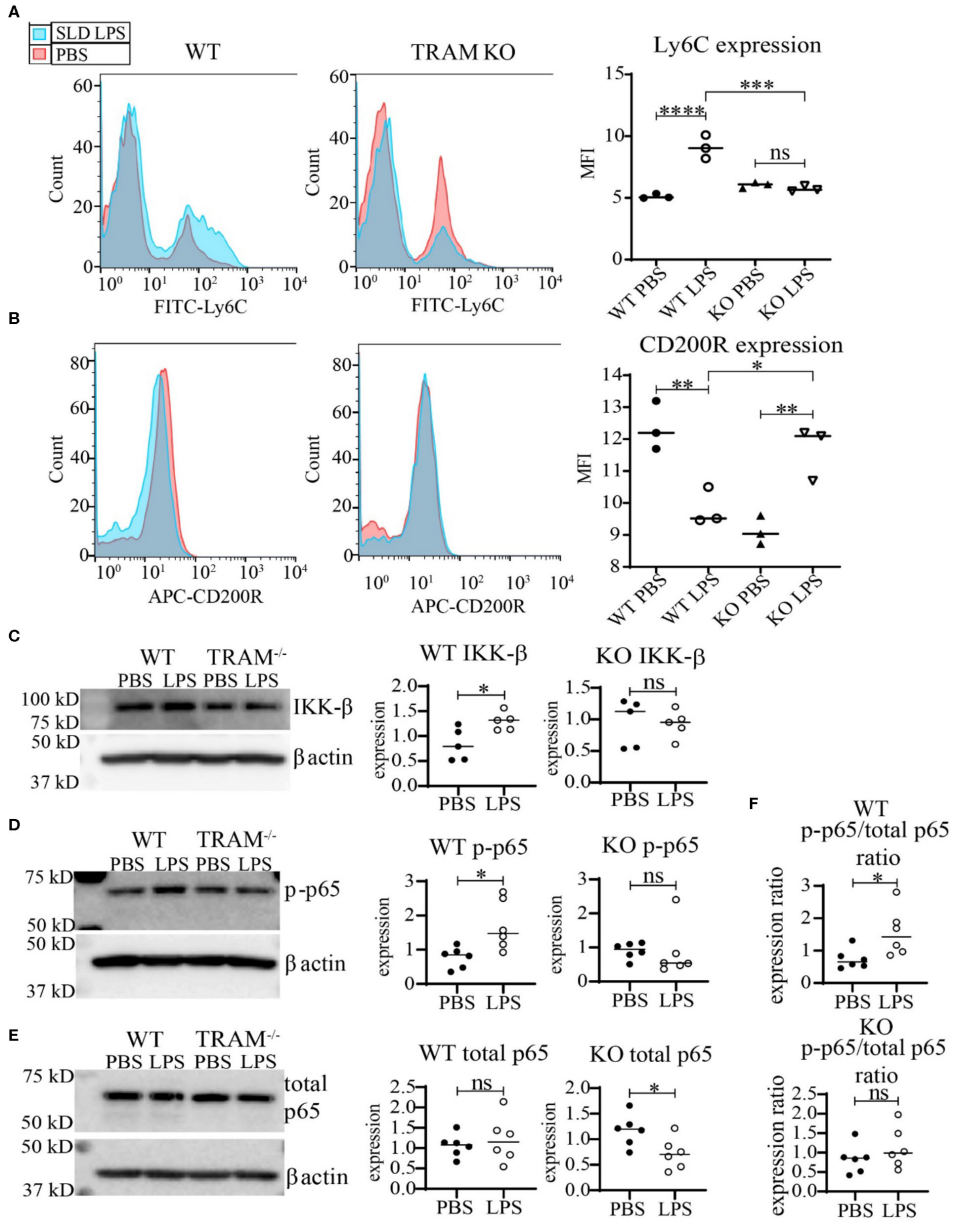
TRAM is required for the pro-inflammatory polarization of macrophages by SLD-LPS. **(A,B)** Flow cytometry analysis of Ly6C **(A)** or CD200R **(B)** expression in WT or TRAM^−/−^ live-cell-gated macrophages with geometric MFI quantification on right. Pink = PBS, Blue = 100 pg/ml LPS. **(C–E)** Representative Western blots of IKK-β **(C)**, p-p65 **(D)**, and total NF-κB p65 **(E)** expression in WT and TRAM^−/−^ monocytes treated with either PBS or 100 pg/mL LPS for 5 days. Quantification is depicted on the right. **(F)** Ratio of p-p65/total p65 (p-p65 levels from **(D)** divided by corresponding total p65 levels). All data are representative of at least 3 separate experiments (*n* = 3) for **(A,B)** and *n* = 6 for **(C–F)**. ANOVA with Tukey's multiple comparisons test **(A,B)**; unpaired student *t*-test **(C–F)** *p < 0.05, **p < 0.01, ***p < 0.001, ****p < 0.0001, n.s, non-significant; MFI, Mean fluorescence intensity.

We further tested whether TRAM deficiency may functionally associate with monocyte inflammatory polarization through measuring the expression of the key cell-surface pro- and anti-inflammatory markers Ly6C and CD200R. As shown in Figure 5, SLD-LPS failed to induce Ly6C in TRAM deficient monocytes as compared to WT monocytes (Figure 5A). On the other hand, SLD-LPS not only failed to suppress the anti-inflammatory marker CD200R in TRAM deficient monocytes, but also significantly induced the levels of CD200R (Figure 5B). Mechanistically, we observed that SLD-LPS failed to induce IKK-β (Figure 5C) or p-p65 (Figure 5D) in TRAM deficient monocytes. Interestingly, while WT monocytes showed no significant difference in total p65 expression, TRAM^−/−^ monocytes showed significant reduction of total p65 protein in response to SLD-LPS (Figure 5E). However, there was no significant difference in the p-p65 to p65 ratio in TRAM deficient monocytes treated with either PBS or SLD-LPS (Figure 5F). Taken together, our data suggest that the TRAM signaling pathway may play a crucial and hitherto un-appreciated role in the non-resolving inflammatory polarization of monocytes by SLD-LPS.

## Discussion

Our current study reveals that murine monocytes persistently challenged with super-low dose LPS can be polarized into a low-grade inflammatory state through the accumulation the TRAM-dependent accumulation of Keap1. First, we documented that chronic SLD-LPS challenge potently induces the accumulation of Keap1 protein in monocytes, in sharp contrast to the previously reported high dose LPS effects of reducing Keap1 (13). Second, we demonstrated that TRAM is critically involved in the SLD-LPS mediated induction of Keap1, as TRAM deficient monocytes fail to accumulate Keap1 following SLD-LPS challenge. Functionally, we demonstrated that TRAM is necessary for the SLD-LPS-mediated expansion of the pro-inflammatory Ly6C^high^, Keap1^high^ monocyte population.

Subclinical endotoxemia is an emerging phenomenon widely documented in experimental animals and human patients with chronic inflammatory conditions (5, 46–48), and may exacerbate the pathogenesis of chronic inflammatory diseases. However, despite its emerging clinical relevance, the effects of SLD-LPS on monocyte polarization and underlying mechanisms are still relatively less studied as compared to high dose LPS. A prolonged challenge with higher dose LPS can reduce the cellular levels of Keap1 (13). The reduction of Keap1 has been causally connected with the activation of Nrf2 (21), consistent with the anti-inflammatory adaptation of monocytes challenged with higher dose LPS. In contrast, our current data reveal that super-low dose LPS can potently increase the cellular levels of Keap1 in monocytes. Our data extend previous studies regarding the mechanistic connection of Keap1 with Nrf2 in the unique setting of monocytes persistently challenged with SLD- LPS. The increase of Keap1 in SLD-LPS polarized monocytes also correlates with the accumulation of p62, MLKL, and IKKβ, all of which are associated with defective autolysosomal fusion (10). This supports our previous confocal studies showing impaired endolysosomal acidification and lysosomal fusion in monocytes treated with super-low doses of LPS (10). This unique mechanism is functionally correlated with the pro-inflammatory polarization of monocytes by SLD-LPS, as reflected in the expansion of the inflammatory Ly6C^high^ monocyte population and the simultaneous reduction of anti-inflammatory CD200R in monocytes. We further demonstrated that Keap1 levels are increased in the sub-population of Ly6C^high^ monocytes.

One potential explanation that may account for the dose-dependent modulation of Keap1 could be the differential usage of distinct TLR4 downstream signaling adaptors. TLR4 signaling can proceed through either MyD88-dependent or TRAM/TRIF-dependent signaling, with the MyD88-dependent pathway being the presumed predominant pro-inflammatory signaling pathway (49–51). However, the majority of these studies have been conducted using high doses of LPS and/or short incubation times. Here we demonstrate that the upregulation of Keap1 and pro-inflammatory polarization of mouse monocytes in response to chronic SLD-LPS is mediated through the TRAM-dependent pathway. Recently it has been shown that this pathway is crucial for the delayed but prolonged NF-κB-mediated inflammatory response (18), possibly contributing to the persistent low-grade inflammation associated with chronic inflammatory diseases such as atherosclerosis and obesity (52, 53). The TRAM/TRIF pathway, but not the Mal/MyD88 pathway, was found to be crucial for atherosclerotic lesion development in a study comparing *Mal*^−/−^, *Tram*^−/−^, and *Trif* ^−/−^ bone marrow transplantation in *Ldlr*^−/−^ recipient mice, further strengthening this link (17). These studies coupled with our observations in chronic SLD-LPS-treated monocytes suggest a previously underappreciated role for the TRAM signaling pathway in the pathogenesis of chronic inflammatory diseases. In addition, our data that SLD-LPS preferentially induces Keap1 through TRAM may guide future studies in defining novel therapeutic targets for treating chronic inflammatory diseases.

Despite these novel observations, we realize that our current limited analysis may only serve as a prelude for much-needed future studies regarding monocyte polarization challenged with subclinical endotoxemia. Decades of previous research efforts have already led to extensive knowledge of monocyte activation dynamics when challenged with higher doses of LPS. With regard to the effects of subclinical super-low dose LPS, similar extensive efforts are warranted in future thematic studies to answer pressing issues such as the time-course needed for the establishment of low-grade inflammatory monocyte polarization; the relative stability and plasticity of polarized monocytes; the comparative analysis of murine and human monocytes; as well as the *in vivo* relevance in diverse inflammatory disease settings. In-depth mechanistic studies are also required to clarify detailed cellular and molecular machineries involved in the regulation of autolysosome function in polarized monocytes. To address these questions, future integrated long term studies with both *in vitro* and *in vivo* experimental systems at single cell levels are warranted. However, our current findings of TRAM-mediated SLD-LPS Keap1 upregulation provide a key mechanistic starting point for these future studies.

## Data Availability Statement

All raw data and datasets supporting the conclusions of this article are available by the authors upon request to any qualified researcher.

## Ethics Statement

The animal study was reviewed and approved by Virginia Tech Institutional Animal Care and Use Committee (IACUC).

## Author's Note

It should also be noted that this manuscript was included as a part of Dr. Rahtes' 2019 Ph.D. dissertation titled Non-resolving pro-inflammatory macrophage polarization by super-low doses of bacterial endotoxin in a delayed publication format set to be available on the Virginia Tech online database in 2021 and referenced here as reference number (54).

## Author Contributions

LL and AR contributed to experimental design and writing, while all experiments and data analysis were performed by AR. LL supervised the study. All authors contributed to the article and approved the submitted version.

## Conflict of Interest

The authors declare that the research was conducted in the absence of any commercial or financial relationships that could be construed as a potential conflict of interest.
