# Supplementary material for: Polarization of Low-Grade Inflammatory Monocytes Through TRAM-Mediated Up-Regulation of Keap1 by Super-Low Dose Endotoxin
Source: Front Immunol. 2020 Jul 16;11:1478. doi: 10.3389/fimmu.2020.01478 (PMC7378438; doi:10.3389/fimmu.2020.01478)
Supplement: Supplementary file 1 [file Presentation_1.pptx]

## Slide 1
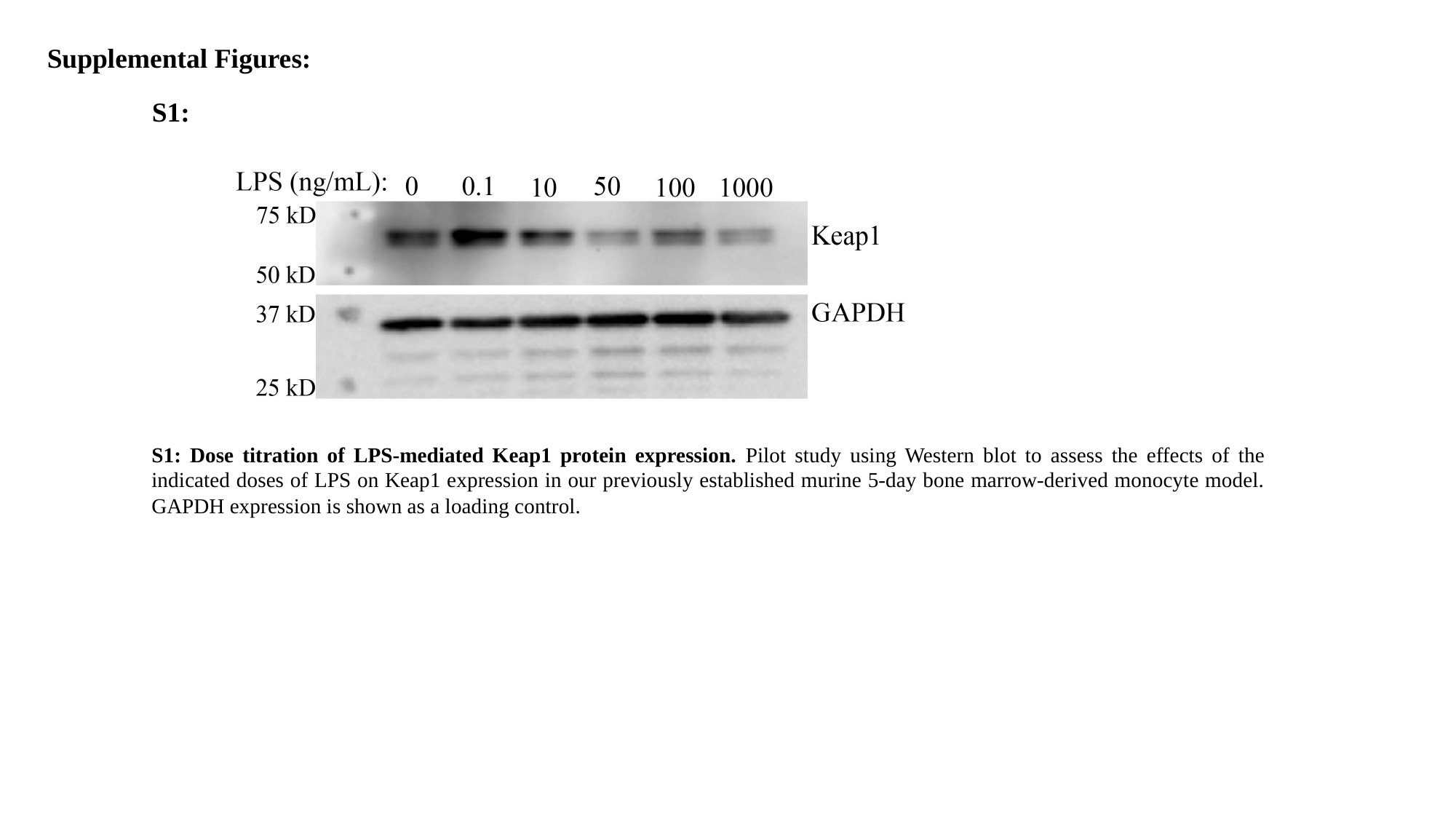

Supplemental Figures:
S1:
S1: Dose titration of LPS-mediated Keap1 protein expression. Pilot study using Western blot to assess the effects of the indicated doses of LPS on Keap1 expression in our previously established murine 5-day bone marrow-derived monocyte model. GAPDH expression is shown as a loading control.

## Slide 2
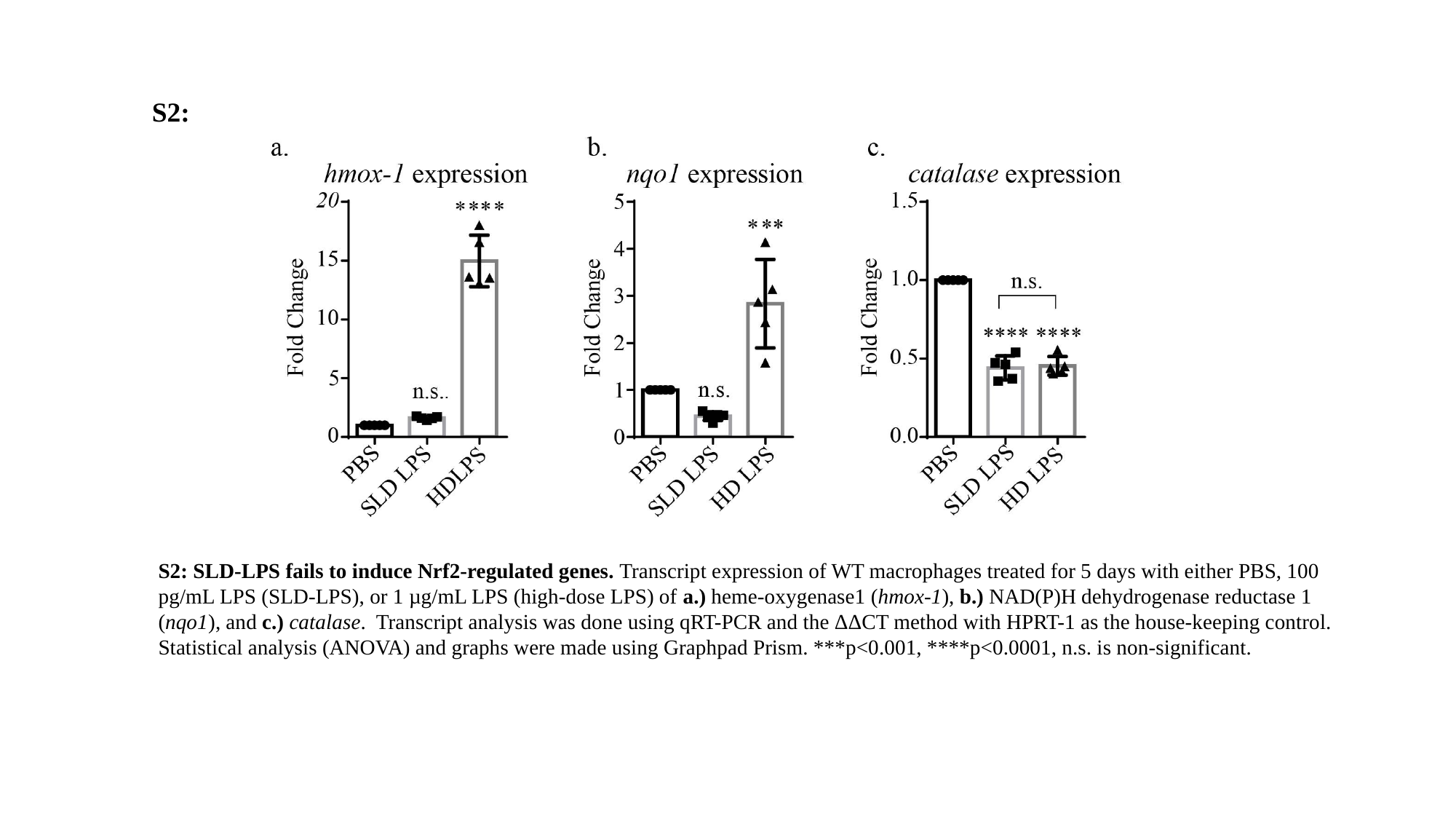

S2:
S2: SLD-LPS fails to induce Nrf2-regulated genes. Transcript expression of WT macrophages treated for 5 days with either PBS, 100 pg/mL LPS (SLD-LPS), or 1 µg/mL LPS (high-dose LPS) of a.) heme-oxygenase1 (hmox-1), b.) NAD(P)H dehydrogenase reductase 1 (nqo1), and c.) catalase. Transcript analysis was done using qRT-PCR and the ΔΔCT method with HPRT-1 as the house-keeping control. Statistical analysis (ANOVA) and graphs were made using Graphpad Prism. ***p<0.001, ****p<0.0001, n.s. is non-significant.

## Slide 3
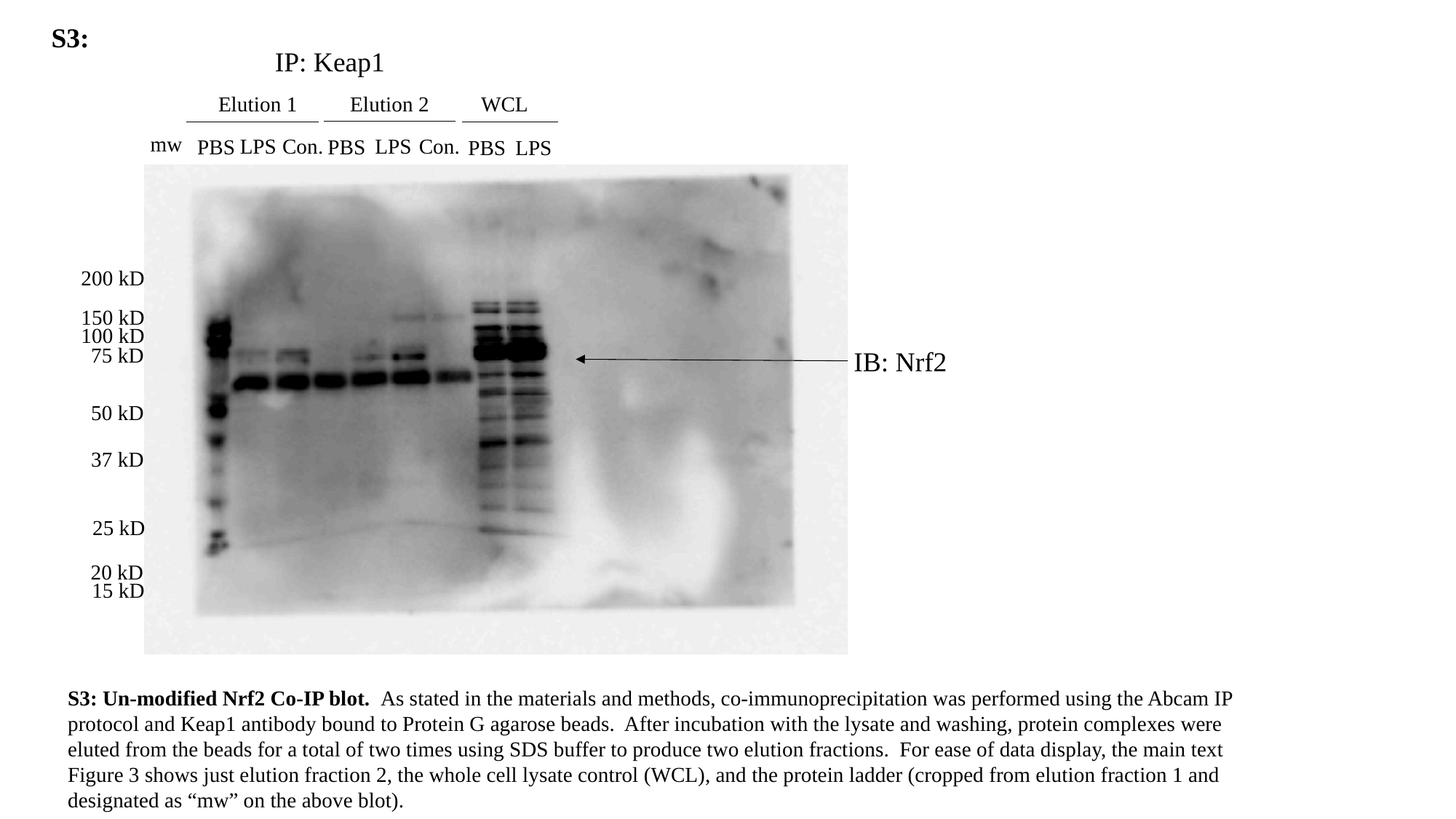

S3:
IP: Keap1
Elution 2
WCL
Elution 1
mw
LPS
LPS
Con.
Con.
PBS
PBS
LPS
PBS
200 kD
150 kD
100 kD
75 kD
IB: Nrf2
50 kD
37 kD
25 kD
20 kD
15 kD
S3: Un-modified Nrf2 Co-IP blot. As stated in the materials and methods, co-immunoprecipitation was performed using the Abcam IP protocol and Keap1 antibody bound to Protein G agarose beads. After incubation with the lysate and washing, protein complexes were eluted from the beads for a total of two times using SDS buffer to produce two elution fractions. For ease of data display, the main text Figure 3 shows just elution fraction 2, the whole cell lysate control (WCL), and the protein ladder (cropped from elution fraction 1 and designated as “mw” on the above blot).

## Slide 4
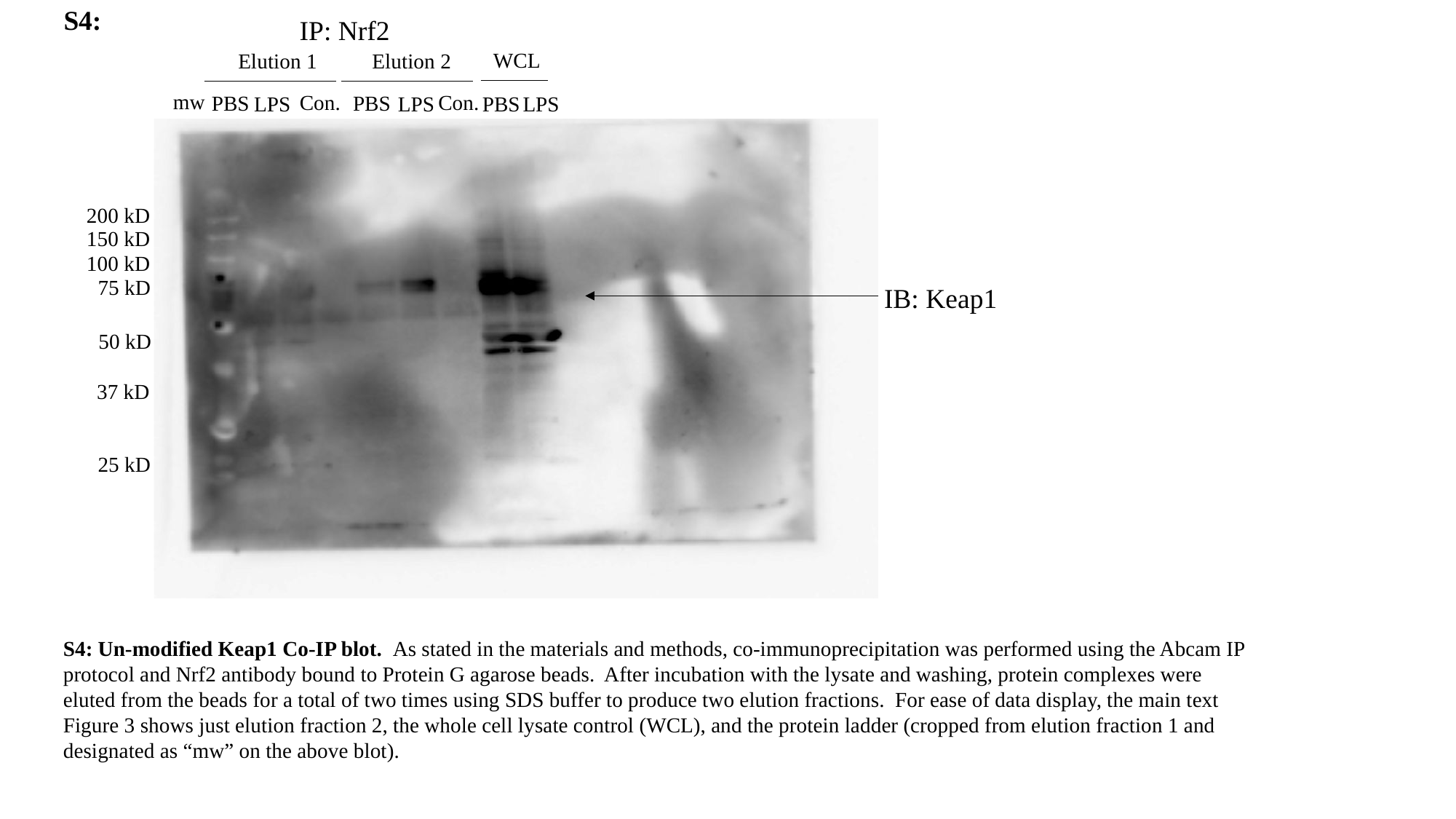

S4:
IP: Nrf2
WCL
Elution 2
Elution 1
mw
Con.
Con.
PBS
PBS
LPS
LPS
LPS
PBS
200 kD
150 kD
100 kD
75 kD
IB: Keap1
50 kD
37 kD
25 kD
S4: Un-modified Keap1 Co-IP blot. As stated in the materials and methods, co-immunoprecipitation was performed using the Abcam IP protocol and Nrf2 antibody bound to Protein G agarose beads. After incubation with the lysate and washing, protein complexes were eluted from the beads for a total of two times using SDS buffer to produce two elution fractions. For ease of data display, the main text Figure 3 shows just elution fraction 2, the whole cell lysate control (WCL), and the protein ladder (cropped from elution fraction 1 and designated as “mw” on the above blot).
